# The effects of mindfulness‐based interventions on nurses' anxiety and depression: A meta‐analysis

**DOI:** 10.1002/nop2.1610

**Published:** 2023-01-24

**Authors:** Hui Liu, Luya Kong, Qian Sun, Xiaofeng Ma

**Affiliations:** ^1^ School of Public health Changsha Medical University Changsha China; ^2^ Psychology of Teaching and Research Section, Ideological and Political Department HZ Domestic Professional College Heze China

**Keywords:** anxiety, depression, meta‐analysis, mindfulness, nurses

## Abstract

**Objective:**

This meta‐analysis aimed to determine the effect of mindfulness interventions on nurses' levels of depression and anxiety.

**Design:**

Meta‐analysis of randomised controlled trials.

**Methods:**

The following Chinese and English databases were searched: PubMed, Embase, Cochrane Library, Web of Science, and China National Knowledge Internet (CNKI). The retrieval period was from database construction to 30 March 2022. Two researchers screened the relevant literature and extracted the data. After a cross‐check, data were input into Stata version 16.0 for meta‐analysis.

**Results:**

Twelve randomised controlled trials from 2017 to 2021 were included, which involved 807 subjects (405 and 402 in the intervention and control groups, respectively). Meta‐analysis results showed that nurses' anxiety reduced by mindfulness‐based interventions was significantly higher compared to that of the control group (SMD = 0.91, 95% CI: 0.27–1.55, *p* < 0.05). Furthermore, an 8‐week mindfulness‐based intervention (SMD = 1.43, 95% CI: 0.61–2.24) reduced the level of anxiety significantly more compared to a 4‐week intervention (SMD = 1.03, 95% CI: 0.36–1.71). Mindfulness‐based interventions were better compared to conventional intervention to reduce the level of depression (SMD = 1.02, 95% CI: 0.42–1.61, *p* < 0.05), and an 8‐week mindfulness intervention (SMD = 1.81, 95% CI: 0.78–2.84) reduced the level of depression significantly more compared to a 4‐week intervention (SMD = 0.82, 95% CI: 0.29–1.35). Since limited studies had interventions longer than 8 weeks, results on longer mindfulness interventions in reducing nurses' anxiety and depression are inconclusive. In conclusion, mindfulness intervention for 8 weeks or less can significantly reduce nurses' anxiety and depression levels.

**Patient or Public Contribution:**

None.

## 1 INTRODUCTION

Anxiety, a feeling of fear, occurs during a threatening or stressful situation, while depression usually involves a low mood, fatigue, and trouble sleeping in the face of stress (Aihong, 2017; Allen et al., 2006). Mild symptoms of anxiety or depression may seem vague; however, a rating scale can determine the severity of the illness. Once diagnosed, they require appropriate treatment (Beerse et al., 2020). Nursing staff have high responsibility, high risk, heavy workload, and often face patients' pain and death. Heavy pressure leaves nurses at a high risk of anxiety and depression (Botha et al., 2015). Anxiety and depression threaten nurses' physical and mental health and also increase the turnover rate and risk of nursing errors, resulting in a decline in nursing quality (Cheung & Yip, 2015).

Originally from the Eightfold Path of Buddhism, mindfulness, a Buddhist practice, emphasises the conscious awareness of the present without judgement. Mindability‐based therapies is an umbrella term for various psychotherapies that focus on mindfulness. Mindfulness‐based interventions include mindfulness‐based stress reduction (MBSR) and mindfulness‐based cognitive therapy (MBCT). First developed by Kabat‐Zinn in 1982, MBSR trains patients with chronic pain in self‐regulation through a 10‐week stress reduction and relaxation program (Chunmei & Chen, 2020). It has since been widely used in stress management. Originally developed by Teasdale JD, MBCT aims to prevent the recurrence of major depression by combining the elements of mindfulness training and cognitive therapy (Chunnan et al., 2021). Mindfulness‐based interventions can effectively reduce a series of individual symptoms for anxiety and depression and their severity (Cumpston et al., 2019). The main implementation method included team teaching by mindfulness therapists to guide the objective of mindfulness training, training content including mindfulness diet, mindful breathing training, mindfulness meditation, mindfulness practice yoga, mindfulness sitting, and mindfulness walking. An intervention exercise record sheet was issued at the end of the mindfulness intervention to record negative emotions and pleasant or unpleasant events. Till date, most studies on the effect of mindfulness interventions on nurses focused on job burnout, and less attention has been paid to nurses' anxiety and depression. Many studies have reported on the systematic evaluations of mindfulness‐based interventions to improve nurses' job burnout (Daigle et al., 2018), stress(Dean, 2016), and mental health (Deyang & Shoumin, 2018); however, there are no evidence‐based reports for the effect of intervention on nurses' anxiety and depression. Therefore, this study conducted a meta‐analysis based on previous literature to explore the effects of mindfulness interventions on nurses' anxiety and depression to provide evidence for its clinical application.

## 2 MATERIALS AND METHODS

This meta‐analysis was designed according to the Preferred Reporting Items for Systematic Reviews and Meta‐Analysis (PRISMA) guidelines (Guendelman et al., 2017).

### 2.1 Inclusion criteria

The inclusion criteria for this meta‐analysis were: (1) Participants: Nurses who had obtained their nurse qualification certificate, worked in clinical practice, and were at least aged 18 years; (2) Intervention: Mindfulness‐based interventions, which included mindful meditation, mindful yoga, mindful cognitive training, and mindfulness‐based stress reduction (MBSR), etc; (3) Comparison: Blank control or routine intervention; (4) Outcome: Changes in nurses' anxiety and depression levels before and after the intervention (unrestricted evaluation scale); and (5) Study: Randomised controlled trials (RCT).

### 2.2 Exclusion criteria

The exclusion criteria were: (1) nursing students; (2) none RCTs; (3) Other types of interventions were used; (4) Full text or data were unavailable or incomplete; (5) Duplicated manuscripts; and (6) Low‐quality literature.

### 2.3 Search strategy

All published articles from the establishment of the database till 30 March 2022 were via the PubMed, Cochrane Library, Embase, Web of Science, and China National Knowledge Internet (CNKI) databases. In addition, the search scope was expanded manually by searching for references to other articles and going to the library to find journals. Using PubMed as an example, the retrieval formula is shown in Table 1. After literature were retrieved, all records were imported into Endnote X9 for classification.

**TABLE 1:** Literature retrieval strategy

Search number	Query
9	#4 AND #5 AND #8
8	#6 OR #7
7	Anxiety*
6	Depression*
5	Nurse
4	#1 OR #2 OR #3
3	(MBSR) OR (MBCT)
2	(mindfulness‐based stress reduction) OR (mindfulness therapy) OR (mindfulness‐based intervention) OR (mindfulness‐based cognitive therapy)
1	Mindfulness*

*represents the subject words in the retrieval strategy.

### 2.4 Literature screening and data extraction

Two investigators independently screened the literature and extracted and cross‐checked the data. Disagreements were resolved through consultation with a third investigator. After obviously irrelevant literature were excluded, the full texts were read for rescreening. When important information or data were incomplete, we contacted the authors by mail or telephone to obtain additional information. The extracted contents included (1) basic information of the study: author(s), publication year, country, duration of the intervention, hospital departments, intervention, sample size, age, female‐to‐male ratio, etc.; (2) outcome measures of interest; and (3) conditions required to assess the risk of bias.

### 2.5 Quality evaluation and statistical analysis

Two researchers used the bias risk assessment tool of RCTs recommended in the Cochrane Handbook 5.1.0 (Guojie et al., 2021) to evaluate the bias risk of the included studies and cross‐check the results. The assessment of bias risk included random sequence generation, allocation concealment, blinding, missing outcome data, and selective reporting of outcomes. Review Manager version 5.3 was used to generate graphic manifestations of potential biases within and across the studies.

Stata version 16.0 was used for statistical analysis. *Q* test and *I* ^2^ statistics were used to test the heterogeneity of the included studies. If *p* < 0.1 and *I* ^2^ > 50%, heterogeneity was considered to exist between studies, and a random‐effects model was adopted. In contrast, a fixed‐effects model was used. Continuous data were represented by the standardised mean difference (SMD) and 95% confidence interval (CI). The results of the meta‐analysis were presented with a forest map. Subgroup and sensitivity analyses were performed to explore the source of heterogeneity in the results with high heterogeneity. Egger's test and funnel plots were used to evaluate publication bias. Statistical significance was set at *p* < 0.05.

## 3 RESULTS

### 3.1 Study characteristics

**FIGURE 1:**
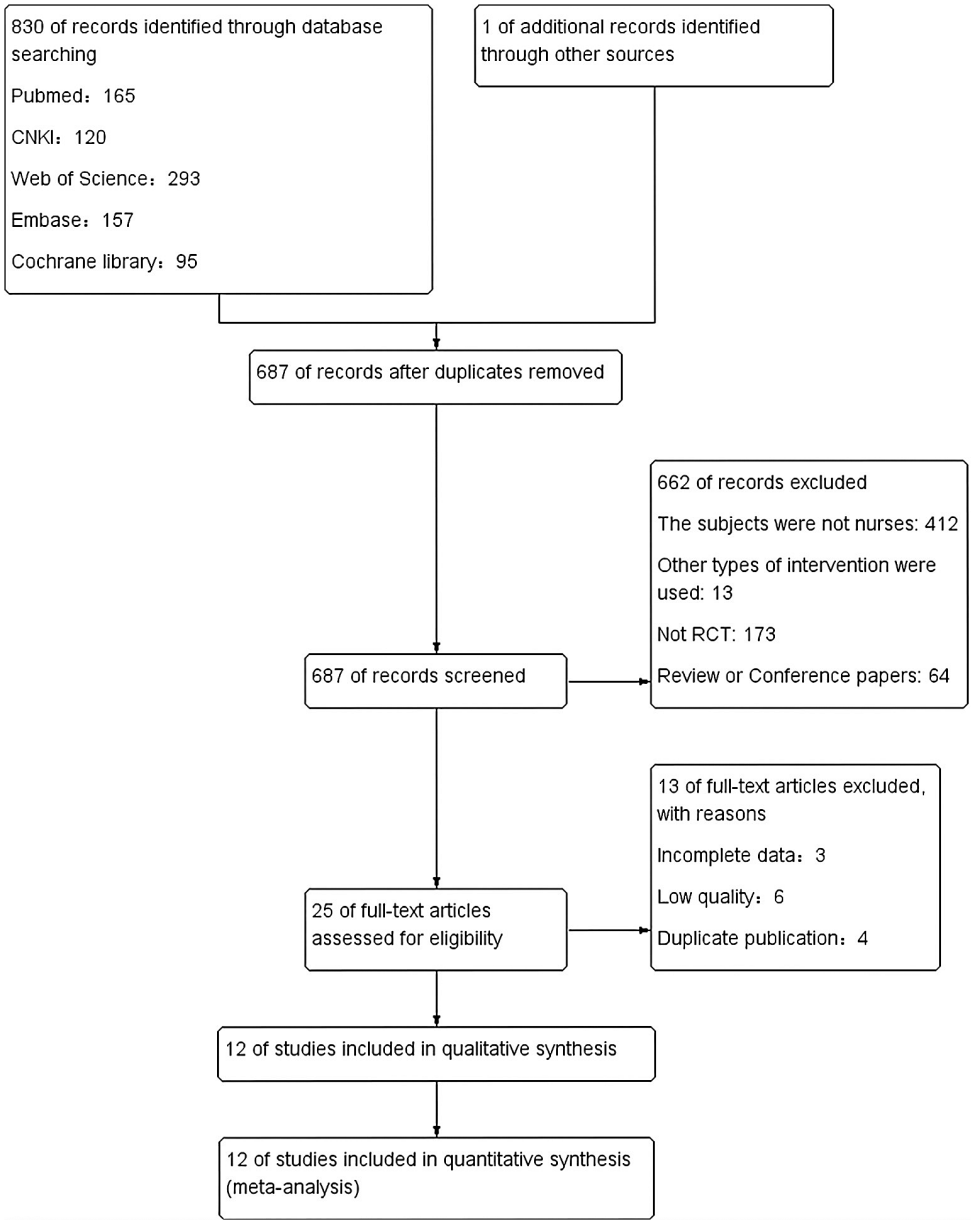
Literature screening flow chart

A total of 831 studies in English or Chinese were retrieved, which included 830 studies in from the databases and one study from another source. Of these, 144 duplicate references were excluded using EndNote X9. After the titles and abstracts were read, another 662 studies were excluded. Furthermore, 13 other studies were excluded after the full text was read. Finally, 12 RCTs were included in this meta‐analysis. The literature screening and exclusion process is presented in Figure 1.

Twelve randomised controlled trials from 2017 to 2021 were included (Hofmann et al., 2010; Hofmann & Gómez, 2017; Kabat‐Zinn, 1982; Kang & Myung, 2022; Liberati et al., 2009; Lihua et al., 2017; Maharaj et al., 2018; Qiu, 2019; Rakel, 1999; Ramachandran et al., 2022; Shapiro et al., 2006; Teasdale et al., 2000), which involved 807 subjects (405 and 402 in the intervention and control groups, respectively). One study was from Canada (Hofmann et al., 2010), one from Japan (Kabat‐Zinn, 1982), and the remaining 10 were from China (Hofmann & Gómez, 2017; Kang & Myung, 2022; Liberati et al., 2009; Lihua et al., 2017; Maharaj et al., 2018; Qiu, 2019; Rakel, 1999; Ramachandran et al., 2022; Shapiro et al., 2006; Teasdale et al., 2000). Of these, three(Hofmann et al., 2010; Hofmann & Gómez, 2017; Kabat‐Zinn, 1982) were published in English and the remaining nine in Chinese. All participants were clinical nurses, interventions were mindfulness interventions for 4–13 weeks, and the control group had a blank control or routine intervention that did not include mindfulness. The outcome measures included anxiety and depression levels. The basic characteristics of the included studies are shown in Tables 2.1 and 2.2.

**TABLE 2.1:** Characteristics of studies included in the meta‐analysis

Study	Year	Country	Duration of intervention	Age		Sex (female/male)	
				Intervention group	Contrast group	Intervention group	Contrast group
Yang J (Hofmann & Gómez, 2017)	2018	China	8 weeks	29.2 ± 6.9		32/16	32/15
Daigle S (Hofmann et al., 2010)	2018	Canada	8 weeks	47.03 ± 9.7	45.30 ± 9.5	–	
Watanabe N (Kabat‐Zinn, 1982)	2019	Japan	13 weeks	30.2 ± 9.0	30.0 ± 7.9	40/0	40/0
Lihua L (Kang & Myung, 2022)	2017	China	8 weeks	–		–	
Chunnan T (Liberati et al., 2009)	2021	China	4 weeks	25.25 ± 5.13	24.94 ± 4.77	14/2	14/2
Chunmei W (Lihua et al., 2017)	2020	China	4 weeks	28.99 ± 8.63		90/10	
Deyang W (Maharaj et al., 2018)	2018	China	4 weeks	30.21 ± 6.35	29.13 ± 5.37	27/3	29/1
Xiumei W (Qiu, 2019)	2018	China	12 weeks	32.6 ± 4.2	33.2 ± 4.0	36/2	36/1
Aihong W (Rakel, 1999)	2017	China	8 weeks	26.5 ± 5.5		30/0	30/0
Qiu J (Ramachandran et al., 2022)	2019	China	4 weeks	24 ~ 45	25 ~ 48	–	
Yaxu W (Shapiro et al., 2006)	2021	China	4 weeks	41 ± 8	40 ± 7	25/2	25/4
Guojie L (Teasdale et al., 2000)	2021	China	8 weeks	28.43 ± 6.59		72/17	

**TABLE 2.2:** Characteristics of outcome indicators in the included literature

Study	Interventions		Sample size		Outcome	Measuring tool
	Intervention group	Contrast group	Intervention group	Contrast group		
Yang J (Hofmann & Gómez, 2017)	MBSR	Blank control	48	47	Anxiety Depression	②③
Daigle S (Hofmann et al., 2010)	MBSR	Usual care	37	33	Anxiety	⑦
Watanabe N (Kabat‐Zinn, 1982)	MBT	Usual care	40	40	Anxiety Depression	⑤
Lihua L (Kang & Myung, 2022)	MBSR	Blank control	30	30	Anxiety Depression	②③
Chunnan T (Liberati et al., 2009)	MBT	Usual care	16	16	Anxiety Depression	②③
Chunmei W (Lihua et al., 2017)	MBSR	Usual care	50	50	Anxiety Depression	②③
Deyang W (Maharaj et al., 2018)	MBSR	Usual care	30	30	Anxiety Depression	②③
Xiumei W (Qiu, 2019)	MBSR	Usual care	38	37	Anxiety Depression	④
Aihong W (Rakel, 1999)	MBSR	Usual care	30	30	Anxiety Depression	②③
Qiu J (Ramachandran et al., 2022)	MBSR	Usual care	15	15	Anxiety	⑥
Yaxu W (Shapiro et al., 2006)	MBSR	Blank control	27	29	Anxiety Depression	①
Guojie L (Teasdale et al., 2000)	MBSR	Blank control	44	45	Anxiety Depression	②③

*Note*: ① Depression, Anxiety, and Stress Scale (DASS); ② The self‐rating depression scale (SDS); ③ The self‐rating anxiety scale (SAS); ④ Symptom Check List‐90 (SCL‐90); ⑤ Hospital Anxiety and Depression Scale (HADS); ⑥ Hamilton Anxiety Scale (HAMA); ⑦ Tension‐Anxiety subscale of the Profile of Mood States: (POMS‐TA).

Abbreviations: MBSR, mindfulness‐based stress reduction; MBT, Mindfulness‐based training.

### 3.2 Quality assessment

**FIGURE 2:**
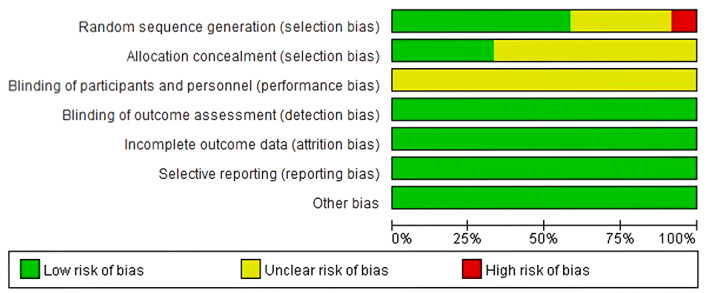
Risk of bias graph

The results of the quality evaluation of the 12 included studies are shown in Figure 2. Random sequence generation methods were reported in seven studies (Hofmann & Gómez, 2017; Kabat‐Zinn, 1982; Kang & Myung, 2022; Liberati et al., 2009; Maharaj et al., 2018; Qiu, 2019; Shapiro et al., 2006), which included the random number table method and random number generator. One study (Teasdale et al., 2000) was grouped according to the order of entry into the study. Four studies (Kang & Myung, 2022; Liberati et al., 2009; Qiu, 2019; Shapiro et al., 2006) reported allocation concealment of a random scheme. Due to the particularity of the study, all mindfulness‐based interventions required the active cooperation of the participants, and the blind principle could not be performed. Therefore, the principle of blind method was not explained in all the studies; however, informed consent from the participants was obtained. Data from all the studies were complete without selective reports, and there were no other deviation risks.

### 3.3 Meta‐analysis results

#### 3.3.1 Anxiety scores

##### Anxiety scores meta‐analysis results

**FIGURE 3:**
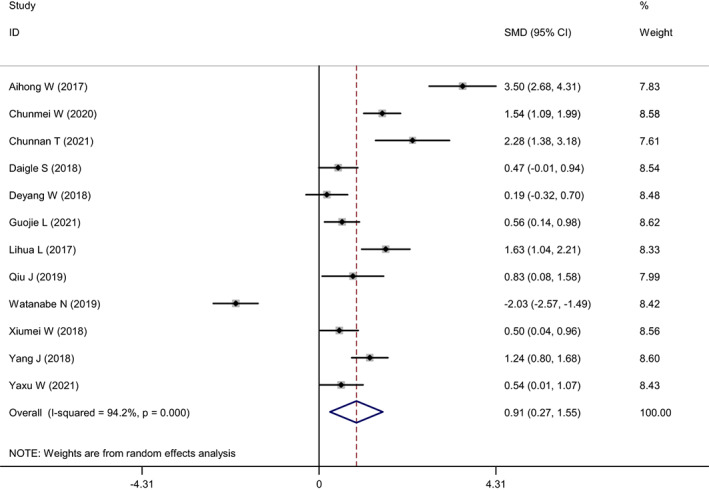
Forest plot of anxiety reduction in the mindfulness intervention group and the control group

**FIGURE 4:**
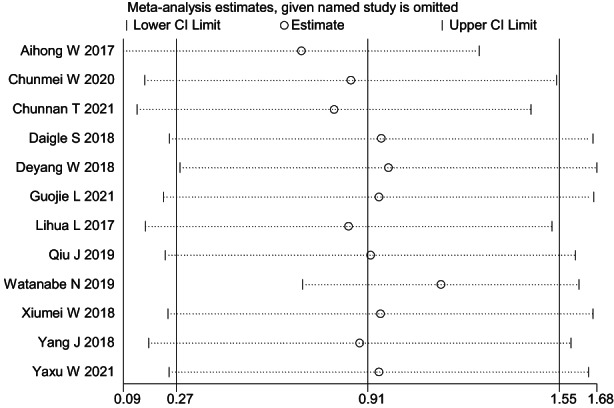
Sensitivity analysis of anxiety reduction in the mindfulness intervention group and the control group

The 12 included studies reported changes in participants' anxiety levels. The heterogeneity test showed *I* ^2^ = 94.2% and *p* = 0.000, which indicated significant heterogeneity between the studies. A meta‐analysis was conducted using random effects model (as shown in Figure 3), and the combined SMD was 0.91 (95% CI: 0.27–1.55). Nurses' anxiety level reduced by mindfulness interventions was significantly higher compared to that of the control group. Furthermore, the difference was statistically significant (*p* = 0.005). A sensitivity analysis was conducted on the included studies to explore the sources of heterogeneity (Figure 4). Regardless of the studies that were excluded, heterogeneity did not decrease significantly, nor did the combined results change significantly, which suggested that the results of this study were relatively robust.

##### Subgroup analysis

**FIGURE 5:**
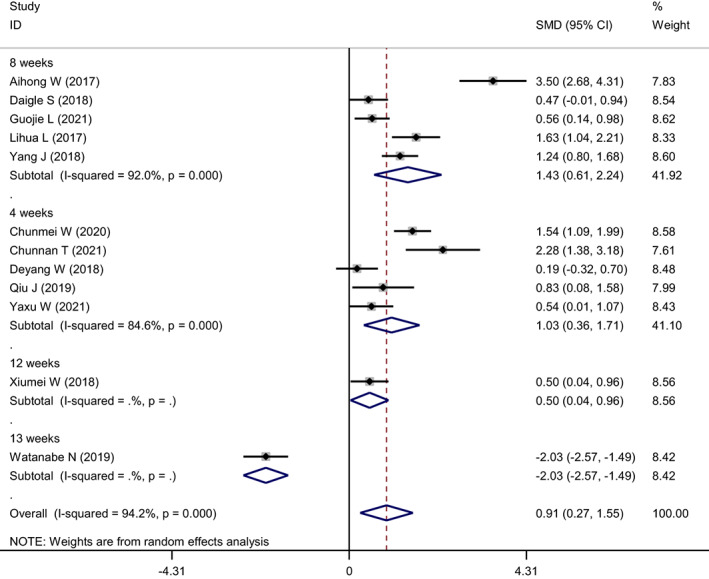
Subgroup analysis forest map of reduced anxiety levels in mindfulness intervention group and control group (grouped by intervention duration)

The intervention duration in the included studies ranged from 4 to 13 weeks, with five that lasted for 8 weeks, five for 4 weeks, one for 12 weeks, and one for 13 weeks. A subgroup analysis was performed based on the intervention time (Figure 5). Heterogeneity was *I* ^2^ = 92.0% (*p* = 0.000) for the 8‐week of intervention (combined SMD = 1.43 (95% CI: 0.61–2.24)). It was *I* ^2^ = 84.6% and *p* = 0.000 for the 4‐week intervention, and the combined SMD of the random effects model was 1.03, 95% CI: 0.36–1.71. Since there was only one study for the intervention duration for 12 weeks and 13 weeks, it could not be combined; therefore, it was not included in the subgroup. The results of the subgroup analysis of intervention duration showed that the longer the intervention time, the more the anxiety levels were reduced.

**FIGURE 6:**
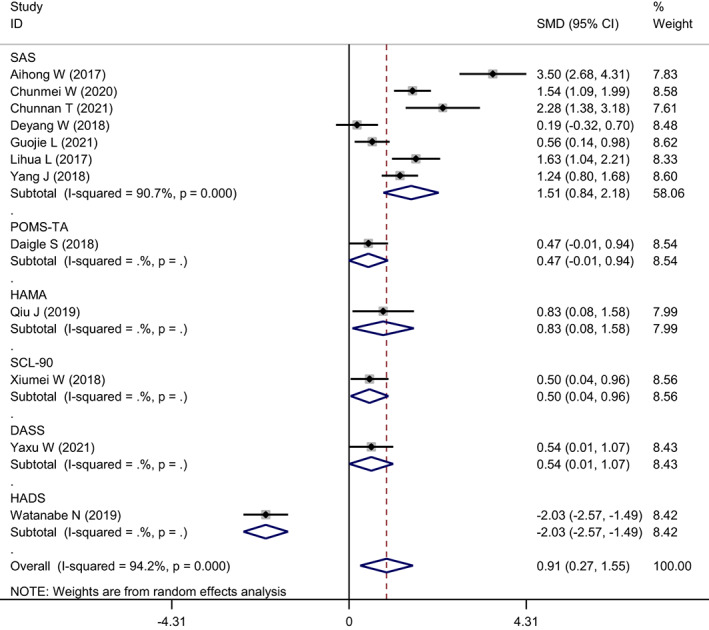
Subgroup analysis forest map of reduced anxiety levels in mindfulness intervention group and control group (grouped by measurement scale)

Six scales were used to evaluate anxiety levels, among which seven studies used the Self‐rating Anxiety Scale (SAS) and one used each of the other scales. The measurement scale was divided into subgroups for analysis (Figure 6). Since there was only one study that used the other scales, subgroups were not included, and only studies that used the SAS were analysed. The heterogeneity of the SAS subgroups was *I* ^2^ = 90.7%, *p* = 0.000, and the random effect model combined SMD = 1.51, 95% CI: 0.84–2.18, *p* = 0.000. The SAS was used to assess the reduction in anxiety by mindfulness intervention more compared to the other scales.

##### Publication bias

**FIGURE 7:**
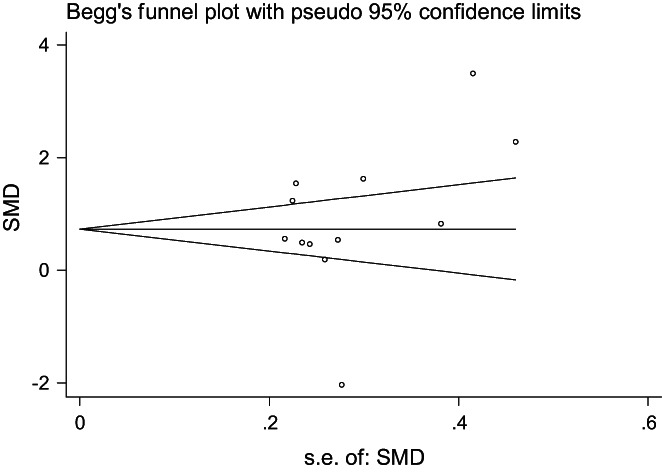
Publication bias of anxiety reduction in the mindfulness intervention group and the control group

Funnel plots were drawn for the included studies to evaluate publication bias (Figure 7). All research points were scattered, with some discrete points, and there might have been publication bias in the subjective evaluation. Egger's test was used for quantitative evaluation (*t* = 1.08, *p* = 0.307), which suggested that there was no publication bias among the studies.

#### 3.3.2 Depression scores

##### Depression scores meta‐analysis results

**FIGURE 8:**
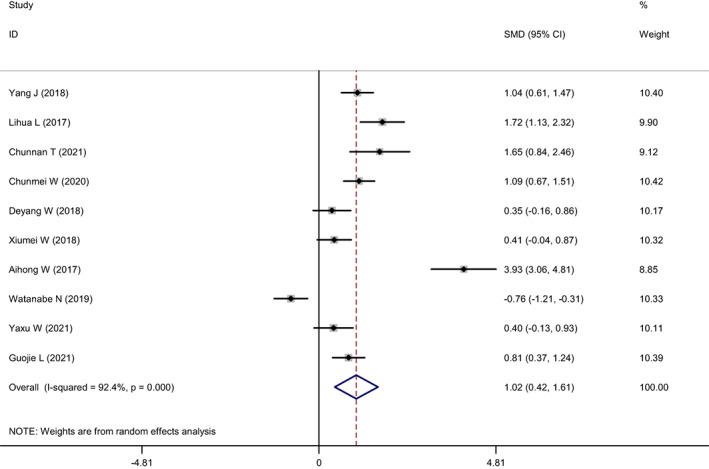
Forest plot of depression reduction in mindfulness intervention group and control group

**FIGURE 9:**
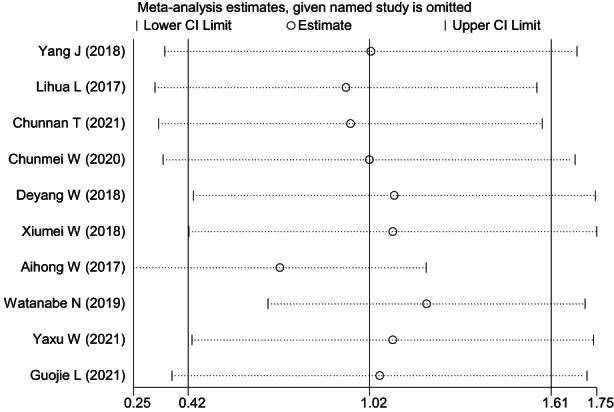
Sensitivity analysis of depression reduction between the mindfulness intervention group and the control group

Ten of the included articles reported changes in participants' depression levels. The heterogeneity test showed *I* ^2^ = 92.4%, *p* = 0.000, which suggested significant heterogeneity between the studies. A meta‐analysis was conducted using the random effects model (Figure 8), and the combined SMD was 1.02, 95% CI: 0.42–1.61. The mindfulness intervention was better compared to the control group in reducing the level of depression, and the difference was statistically significant (*p* = 0.001). Sensitivity analysis was conducted on the included studies to explore the source of heterogeneity (Figure 9). Regardless of the studies that were excluded, heterogeneity did not decrease significantly, and the combined results were all within the credibility range, which suggested that the results of this study were relatively robust.

##### Subgroup analysis

**FIGURE 10:**
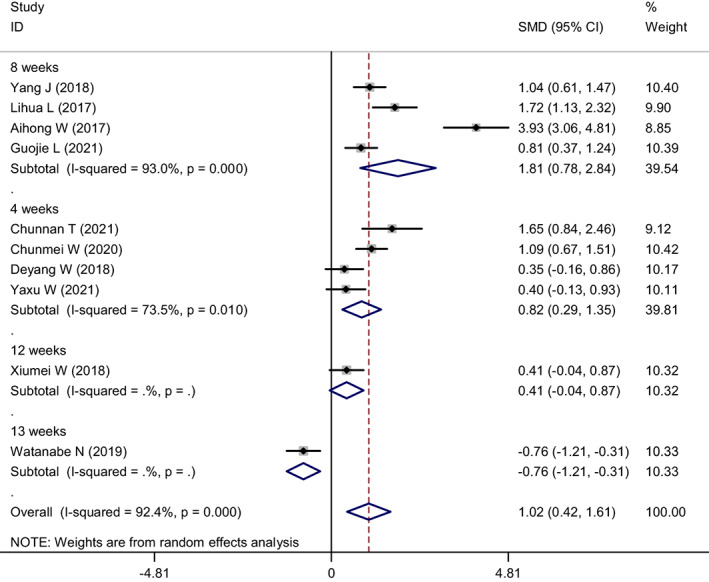
Subgroup analysis forest plot of depression reduction in mindfulness intervention group and control group (grouped by intervention duration)

In the included literature, four studies had an intervention duration of 8 weeks, four with 4 weeks, and one each for 12 and 13 weeks. A meta‐analysis was conducted by subgroup based on intervention time (Figure 10). The heterogeneity of the 8‐week intervention was *I* ^2^ = 93.0% and *p* = 0.000, and the combined SMD of the random effects model was 1.81, 95% CI: 0.78–2.84. It was *I* ^2^ = 73.5% and *p* = 0.010 from the 4‐week intervention, and the combined SMD of the random‐effects model was 0.82, 95% CI: 0.29–1.35. Since there was only one study each for the intervention time of 12 and 13 weeks, it could not be combined; therefore, it was not included in the subgroup. Subgroup analysis of intervention duration showed that the 8‐week mindfulness intervention significantly reduced the level of depression compared to the 4‐week intervention.

**FIGURE 11:**
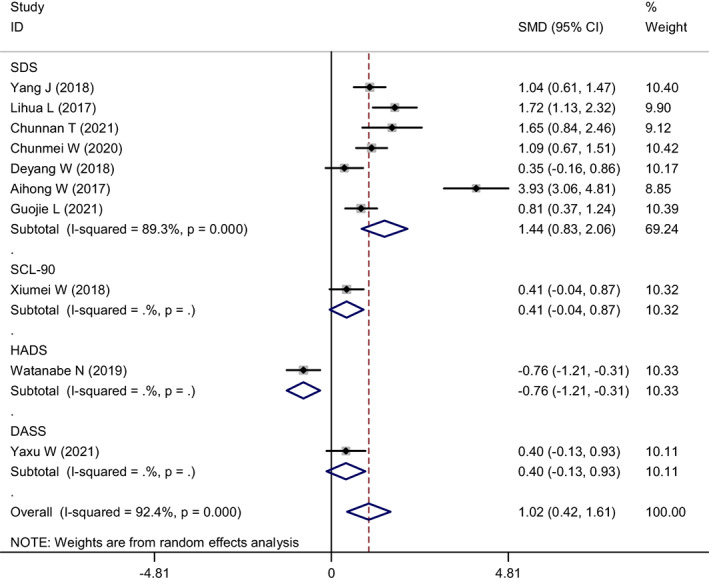
Subgroup analysis forest plot of depression reduction in mindfulness intervention group and control group (grouped by measurement scale)

Four scales were used to assess depression levels, among which seven studies used the Zung Self‐Rating Depression Scale (SDS) and one used each of the other scales. A meta‐analysis was conducted using subgroups of the evaluation scale (Figure 11). Since the Symptom Checklist 90 (SCL‐90), Hospital Anxiety and Depression Scale (HADS), and Depression Anxiety Stress Scales (DASS) were used in only one study, subgroups were not included, and only SDS were analysed. The heterogeneity of SDS subgroups was *I* ^2^ = 89.3% and *p* = 0.000. Furthermore, a meta‐analysis was performed using the random effect model, SMD = 1.44, 95% CI: 0.83–2.06, which suggested that nurses' depression levels, as assessed by the SDS, were reduced.

##### Publication bias

**FIGURE 12:**
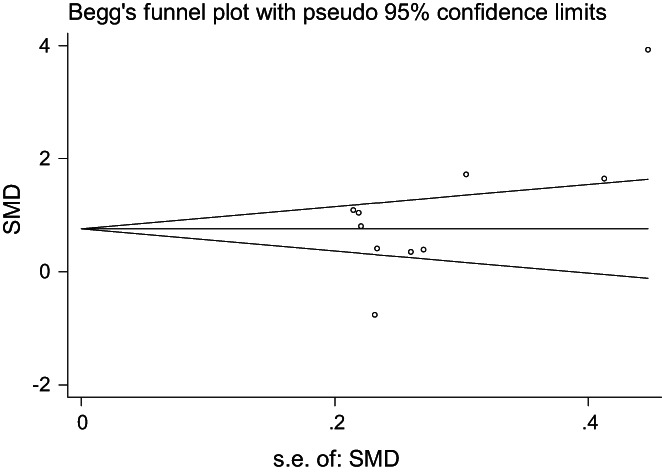
Publication bias of depression reduction between the mindfulness intervention group and the control group

Funnel plots were drawn for the included studies to evaluate publication bias (Figure 12). The distribution of each study point was relatively uniform; however, there were two discrete points, which may have resulted in publication bias. Egger's test was used for quantitative evaluation (*t* = 2.18, *p* = 0.060), which suggested that there was no publication bias between studies.

## 4 DISCUSSION

In this study, 12 high‐quality randomised controlled trials (RCTs), which involved 807 clinical nurses from three countries, were obtained and included in the meta‐analysis from the Chinese and English databases. Published studies on the reduction of nurses' anxiety or depression with mindfulness interventions increased the applicability and credibility of this study. Through meta‐analysis and subgroup analysis, this study found that mindfulness intervention could significantly reduce the anxiety and depression levels of nurses, and the mindfulness intervention duration of 8)weeks had a better effect on reducing the anxiety and depression levels of nurses than the intervention duration of 4)weeks.

The study found that mindfulness‐based intervention significantly reduced anxiety and depression levels among nurses compared with conventional intervention or a blank control group. Mindfulness‐based interventions, the practice of mindfulness (for example, through mindfulness‐based stress reduction, sit‐down meditation, yoga, or other mindfulness practices), causes individuals to be less reactive to unpleasant internal phenomena and more reflective of themselves, resulting in positive psychological responses (Tiller, 2013). Previous studies confirmed that mindfulness‐based interventions reduced the severity of anxiety and depressive symptoms in individuals who had extensively sought treatment (Chunmei & Chen, 2020). A meta‐analysis review based on 39 studies with 1140 participants also found that mindability‐based therapies were moderately effective in improving anxiety and mood disorders (Watanabe et al., 2019). Furthermore, this study found that mindability‐based interventions were equally effective for nurses' anxiety and depressive symptoms. Based on neurobiology, mindfulness intervention may produce biological mediators with positive effects, which can improve sleep quality, reduce salivary cortisol, and change brain structure and function. This can improve mood regulation, self‐control, and stress reduction in intervention subjects (Xiumei & Yanbin, 2018). Psychologically, Shapiro (Yang et al., 2018) proposed a mindfulness model to explain the underlying mechanism of how mindfulness had a positive effect, which suggested that mindfulness worked by changing attention, intention, and attitude. Guendelman (Yaxue et al., 2021) proposed that the nature and mechanism of mindfulness interventions were very complex, and they contained various psycho‐neurocognitive models.

According to the subgroup analysis of the intervention duration, the 8‐week mindfulness intervention reduced anxiety and depression levels better compared to the 4‐week intervention. However, the intervention durations of the included studies ranged from 4 to 13 weeks. Since only one study each had an intervention duration of 12 weeks (Qiu, 2019) and 13 weeks (Kabat‐Zinn, 1982) was insufficient for the meta‐analysis. Hence, additional long‐term intervention and follow‐up studies are required to explore the long‐term impact of mindfulness intervention on nurses' anxiety and depression levels. Seven evaluation scales were used to measure the anxiety or depression levels of nurses in the included studies. Since the SCL‐90 (Qiu, 2019), HADS (Kabat‐Zinn, 1982), DASS (Shapiro et al., 2006), HAM‐A (Ramachandran et al., 2022), and PAMS‐TA (Hofmann et al., 2010) scales were used in only one study, they were not sufficient for the meta‐analysis. A meta‐subgroup analysis of studies that used the SAS and SDS showed that the level of anxiety decreased more with the SAS, and depression decreased more with the SDS. Among all the included studies, Watanabe (Kabat‐Zinn, 1982) used the HADS to assess anxiety and depression levels for 13 weeks; however, the results were not consistent with those of other studies. The author did not find any advantage of mindfulness intervention in reducing anxiety and depression compared to the control group, which suggested that more high‐quality studies were required to confirm this in the future. According to the results of the subgroup analysis by scale, apart from Watanabe's study, mindfulness intervention can effectively reduce nurses' anxiety and depression, regardless of the scale used for the evaluation.

In summary, this study found that mindfulness intervention can significantly reduce the level of anxiety and depression of nurses, suggesting that for clinical nurses with high pressure, mindfulness intervention measures can be reasonably arranged according to their level of anxiety or depression, so as to reduce the occupational anxiety and depression of nurses. This study had some limitations. Firstly, due to the particularity of this study, none of the studies in this meta‐analysis emphasised the principle of blindness, which would affect the objectivity of the results to a certain extent. Thus, this could have affected the results of this study. Second, due to the limited number of relevant studies, most studies included in this review were from China, and only two were from Canada and Japan. The insufficient scope of the included studies may increase the selection bias of this study. Hence, high‐quality studies related to various countries should be included in the future to further verify these results. Finally, according to the intervention time subgroup analysis, the intervention time may be a mindfulness intervention to reduce nurses' level of anxiety or depression. However, the intervention study for a longer time in this study was less, and there were limited research interventions for more than 8 weeks. Hence, mindfulness interventions to reduce nurses' anxiety and depression in the long‐term should be further discussed.

## 5 CONCLUSION

Mindfulness‐based intervention can effectively reduce nurses' level of anxiety and depression. Furthermore, the effect of an 8‐week intervention was better compared to that of a 4‐week intervention.

## AUTHOR CONTRIBUTIONS

H.L. and L.K. wrote the first draft of the paper and had equivalent contribution; L.K. and X.M. designed the research; Q.S. and L.K. provided statistical guidance and revised the manuscript. L.K. acquisition and interpretation of data. H.L., X.M. and Q.S. performed the statistical analysis; All authors approved the final manuscript.

## FUNDING INFORMATION

This subject was supported by Outstanding Youth Project of Hunan Province (No. 20B070).

## CONFLICT OF INTEREST

This paper has no financial interest in any person or organisation.

## ETHICS STATEMENT

This study is a meta‐analysis and does not involve personal samples, so no Ethical approval is required. That is, it is not applicable to this study.

## Data Availability

The datasets used and/or analysed during the current study are available from the corresponding author on reasonable request.
